# The power of integrating data: advancing pain research using meta-analysis

**DOI:** 10.1097/PR9.0000000000001038

**Published:** 2022-10-04

**Authors:** Joel Fundaun, Elizabeth T. Thomas, Annina B. Schmid, Georgios Baskozos

**Affiliations:** a Nuffield Department of Clinical Neurosciences, University of Oxford, Oxford, United Kingdom; b Nuffield Department of Primary Care Health Sciences, Centre for Evidence-Based Medicine, University of Oxford, Oxford, United Kingdom

**Keywords:** Meta-analysis, Random-effects, Common-effect, Fixed-effect, Meta-regression, Network, Individual participant data, Prevalence

## Abstract

Publications related to pain research have increased significantly in recent years. The abundance of new evidence creates challenges staying up to date with the latest information. A comprehensive understanding of the literature is important for both clinicians and investigators involved in pain research. One commonly used method to combine and analyse data in health care research is meta-analysis. The primary aim of a meta-analysis is to quantitatively synthesise the results of multiple studies focused on the same research question. Meta-analysis is a powerful tool that can be used to advance pain research. However, there are inherent challenges when combining data from multiple sources. There are also numerous models and statistical considerations when undertaking a meta-analysis. This review aims to discuss the planning and preparation for completing a meta-analysis, review commonly used meta-analysis models, and evaluate the clinical implications of meta-analysis in pain research.

## Abstract 2

Meta-analysis is a powerful statistical method to quantitatively synthesise data related to pain to ultimately improve patient management and progress future pain research.

## 1. Introduction

The field of pain research has grown substantially in recent years.^53,93^ The rapid increase in research output creates an important need to synthesise these findings. One commonly used tool to combine and analyse data in health care research is using meta-analysis. Often combined with a systematic review of the literature, a meta-analysis aims to quantitatively synthesise the results of multiple studies that answer the same research question.^37,41,76^ Meta-analyses help to understand what is currently known, identify gaps in the literature, and formulate new research questions.

Systematic reviews and meta-analyses are considered the pinnacle of evidence-based medicine.^8,12^ High-quality meta-analyses can guide clinical decision making, inform national and international clinical guidelines, update health care policies, and influence research priorities and funding. This is relevant for both clinicians and scientists and can include a variety of pain-related research topics. Examples range from understanding treatments effects in preclinical models of painful conditions,^54,55,95^ measuring the efficacy of physiotherapy^17,26,30^ or pain medications,^21,28,64^ to assessing associations of functional magnetic resonance imaging to placebo treatment.^96,97^

Rigorous meta-analyses have the potential to provide important insights for pain research.^40^ However, aggregating data to answer impactful clinical questions can be challenging.^8^ There are inherent difficulties when combining data sets and selecting the most appropriate statistical method for a meta-analysis.^27,52^ The overall aims of this review are to discuss the preparatory considerations for completing a meta-analysis, review commonly used meta-analysis models, and evaluate the clinical implications of meta-analysis in pain research.

## 2. Planning and design

**Figure 1.**
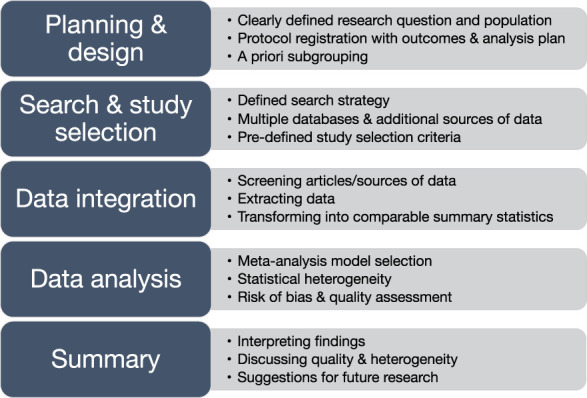
Considerations for completing a systematic literature review with meta-analysis.

Detailed planning and preparation are critical to avoid common pitfalls when conducting a meta-analysis. Methodological errors include poorly designed search strategies, analysing overly dissimilar data, synthesising poor-quality studies, and changing outcomes without properly reporting.^19,27^ These pitfalls can lead to misinterpretation and inaccurate conclusions of the literature. A detailed prospectively registered protocol provides transparency and can mitigate these errors thus strengthening the results and allowing for further scrutiny from the scientific community. Preregistration of a meta-analysis protocol (before completion of data extraction) in an academic journal or an online repository, such as PROSPERO^67^ or the Open Science Framework,^22^ is now a requirement for publication in most high-quality journals. Figure 1 highlights a brief summary of the steps and considerations for undertaking a meta-analysis.

For both systematic reviews and meta-analyses, there are several guidelines to help standardise the study design and reporting of results (eg, Cochrane Handbook,^37^ PRISMA guidelines,^65^ prospective meta-analysis,^77^ preclinical systematic reviews^79^). The EQUATOR Network (https://www.equator-network.org) and CAMARADES (https://www.ed.ac.uk/clinical-brain-sciences/research/camarades) are excellent resources for clinical and preclinical reporting guidelines, respectively. There are also many tools to assess the quality of studies included in a meta-analysis (eg, GRADE,^4^ Cochrane Risk of Bias tool,^36^ Newcastle–Ottawa Scale,^80^ etc). The overall quality and subsequent impact of a meta-analysis can significantly improve through proper study design and appropriate planning.

## 3. Meta-analysis models

There are numerous statistical models to consider when completing a meta-analysis. This review does not provide a comprehensive overview of all available options but will discuss commonly used models in pain research. In each section, we will introduce the model, discuss relevant considerations, and present an illustrative example related to pain research. Table 1 describes a summary of the models discussed in this review. For comprehensive details regarding meta-analysis model application, selection, and statistical methods, refer previous studies.^3,33,34,76^

**Table 1:** Summary of meta-analysis models and corresponding statistical considerations.

Meta-analysis models	Main aim	Considerations
Common-effect	Synthesises the common effect measure between studies	Strengths: Estimates the assumed common underlying treatment effect between studies May be more appropriate for meta-analysis with few included studies Limitations: Problematic when combining the effects of multiple studies because maintaining the assumption that there is no other variance is unlikely Interpretation of results is focused only on the included population
Random-effects	Synthesises the average effect measure between studies	Strengths: More generalisable and less restrictive than the common-effect model Considers heterogeneity of included studies More likely to fit the sampling distribution Limitations: A small number of studies may overinflate effect size estimation High levels of heterogeneity can limit the representation of the identified effect
Meta-regression	Explores potential associations and relationships between studies	Strengths: Assesses strength and direction of relationships Ability to assess multiple covariates simultaneously Limitations: Requires adequate number of studies Must limit covariates based on background subject knowledge
Multivariate	Simultaneously analyses multiple outcomes from the included studies	Strengths: Useful when analysing multiple main outcomes Produces a summary statistic for each outcome Reduces the impact of reporting bias by allowing inclusion of more data Limitations: Correlations measured across studies may not reflect the underlying association between treatment effects Correlation estimates can be less precise and prone to large bias
Network	Assesses available interventions for a clinical condition and makes direct and indirect comparisons across studies to determine the most effective interventions	Strengths: Beneficial for clinicians to decide on the best treatment for patients who fit the review question Limitations: Assumes that heterogeneity variance across different comparisons within the network meta-analysis model is the same Transitivity and inconsistency must be assessed and addressed
Individual participant data	Summarises original data taken from individual participants from multiple studies	Strengths: Increased power to detect differential treatment effects across individuals in randomised controlled trials Ability to identify confounding factors in observational studies Limitations: Difficult to coordinate and obtain individual data Increased time and resource requirements in order to complete
Prevalence	Used to estimate the frequency of a disease occurring within a predefined population	Strengths: A useful tool for clinicians, researchers, and policymakers to better understand the burden of disease Limitations: Variation in the underlying population, case definition, and disease severity is likely to contribute to heterogeneity in the results Transformation of the prevalence proportions may be necessary to obtain confidence intervals that do not lie in extreme ranges and variances that do not result in the undue weighting of studies

Combining data for a meta-analysis focuses on creating an overall effect size estimate of improved precision. An effect size is a quantification of the relationship between 2 entities that incorporates both its direction and magnitude (eg, standardised mean difference, odds, and risk ratios).^37^ It is important to consider the weighting of individual study estimates to improve the precision of the overall estimate for the true effect size of pooled data. The most common method for calculating study weight for continuous data is the inverse-variance method.^9,37^ This method uses the inverse of the variance of the effect size estimate (ie, one over the square of its standard error) to determine the weight given to each study.^9,29^ Similarly, there are other approaches available to calculate the study weight of binary data (eg, Mantel-Haenszel,^74^ Peto^94^). However, determining the most appropriate model to calculate study weight for a meta-analysis remains controversial.^9,33,76^

**Figure 2.**
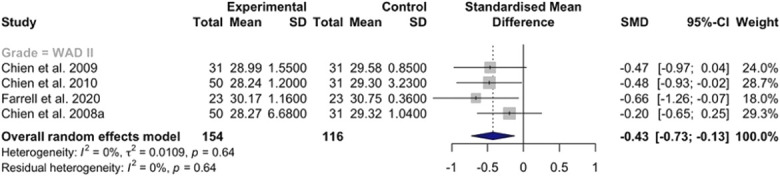
Example forest plot of cold detection thresholds taken at the index finger in patients with whiplash associated disorder (WAD) compared with control subjects. A random-effects model was used to account for potential between-study variance. The left side of the figure displays the total number of participants and corresponding means/standard deviations (SD) for cold detection thresholds of the WAD and control groups. Individual study standardised mean differences (SMD) are depicted by the grey squares (varying in size depending on study weight). The black lines extending from the squares represent the 95% confidence intervals (CI). The overall random-effect meta-analysis summary is shown in bolded text and blue diamond. The overall effect size estimate (blue diamond) does not cross the zero line, indicating that cold detection thresholds were significantly decreased in the WAD groups compared with the control group (*P* < 0.05). The individual and overall SMD, CI, and corresponding study weight values are shown on the right side of the forest plot. Between-study heterogeneity values (shown as Higgins I^2^ and τ^2^) were low and not considered important. This figure was originally published by Fundaun et al.^23^

Effect sizes are graphically depicted using forest plots. Forest plots include critical components of a meta-analysis, including the type of model used, results and weighting of individual studies, the overall effect sizes, confidence intervals, and between-study heterogeneity. Figure 2 includes a detailed description of an example forest plot from our previous systematic review and meta-analysis.^23^

### 3.1. Common-effect model

The common-effect model, also known as the fixed-effect model, is a meta-analysis method that assumes that all included studies share a common effect.^9^ This implies that there is only one true underlying effect (in both magnitude and direction), and the between-study differences are only the result of sampling error, the within-study variance.^9^ For example, this may be applicable when analysing multiple groups (data sets) from a large study performed by a single research group on the same population of individuals and similar experimental paradigms.^9^

#### 3.1.1. Considerations

A common-effect model is applicable if heterogeneity, between-study variance, is not present or when the distribution of the intervention effects is nearly symmetrical.^37^ However, another consideration when choosing between common and random-effect models is accounting for the number of included studies. A small number of studies could overinflate the effect size estimation for random-effects models.^9^ Thus, a common-effect model may produce more robust estimators when comparing only a small number of studies. Conversely, a common-effect model in the presence of heterogeneity can lead to an underestimation of the confidence interval's width because the between-study variance is not taken into account.

#### 3.1.2. Example

Veluchamy et al. performed meta-analyses investigating the association of genetic variants on the susceptibility to neuropathic pain.^91^ The authors performed meta-analyses of genome-wide association studies from 3 large comparable cohorts of patients with neuropathic pain in the United Kingdom. Using common-effect meta-analyses of each single-nucleotide polymorphism,^60^ they identified a novel genome-wide significant locus at chromosome 12q23.1 mapping to SLC25A3 (odds ratio = 1.68, 95% confidence interval [CI]: 1.40–2.02). Experimental models have suggested that SLC25A3 is believed to have a role in developing neuropathic pain; however, further research is required to better understand the underlying mechanisms implicated with these findings.^91^ This study illustrates the use of common-effect models to provide important insights into the potential genetic associations to neuropathic pain.

### 3.2. Random-effects model

In contrast to the common-effect model, the random-effects model allows for the distribution of the true effect size, ie, different effect estimates for each study, and considers additional levels of variance.^9^ In many instances, it is difficult to assume that all studies included in a meta-analysis share one underlying effect size. For instance, studies may measure the same biomarker for a painful condition, but they could have variation in the duration of diagnosis, the timepoint of biomarker analysis, or differences in the type of analytic platform used. Therefore, a random-effects model may be more appropriate because it considers both the within-study and between-study variance (heterogeneity).

#### 3.2.1. Considerations

Random-effects meta-analysis models estimate the variance of the true effect size distribution, which is known as tau^2^.^3,9^ There are multiple methods described to estimate tau^2^. Examples of tau estimators include the restricted maximum likelihood,^92^ DerSimonian–Laird,^18^ Paule–Mandel,^66^ or Sidik–Jonkman.^78^ There is still dispute regarding which estimator is most appropriate.^42,44,51^ In the bias-variance trade-off context, a random-effect meta-analysis is less biased than a common-effect analysis, but it can produce estimators with more variance.

Tau^2^ is necessary to calculate the pooled effect size and indicates the between-study variance. However, tau^2^ does not describe the source of the heterogeneity present between the studies. The quantification of heterogeneity is commonly expressed through measures, including Cochrane Q, showing the variation excess to sampling error, and Higgins I^2^ statistic, showing the excess percentage of the observed Q vs the expected Q.^34,35^ These measures help to understand the extent of between-study heterogeneity present within a meta-analysis but do not identify its source. One method to explore the source of heterogeneity is through subgroup analysis and meta-regression, as discussed below.^90^

#### 3.2.2. Example

Georgopoulos et al.^25^ performed a systematic review and meta-analysis to determine whether quantitative sensory testing (QST) parameters were prognostic of pain and disability in various musculoskeletal conditions. Taken from 37 studies, random-effect meta-analyses identified initial QST measures as prognostic for pain (mean r = 0.31, 95% CI: 0.23–0.38, n = 1,057 participants) and disability (mean r = 0.30, 95% CI: 0.19–0.40, n = 290 participants). This study highlights the potential impact of using QST as a prognostic tool to stratify patients with musculoskeletal pain.

### 3.3. Meta-regression

The interpretation of a meta-analysis is often limited due to potential confounding variables from combining studies. One way to “explore” the potential associations and relationships between the studies, while controlling for covariates, is using meta-regression.^2^ Like linear regression, meta-regression evaluates whether there is a linear relationship between the variables using weighted summary statistics from the included studies. Meta-regression evaluates both the strength and direction of association between the covariates within an analysis.^2^

#### 3.3.1. Considerations

Common-effect meta-regression models do not consider between-study variance, making random-effects models more appropriate for meta-regression.^7,84^ Compared with subgroup analysis, meta-regression provides more detailed consideration for the strength and direction of relationships between the covariates. The selection of covariates (eg, age, sex, comorbidities, etc) should be limited in number, based on background subject knowledge, and should be determined a priori.

#### 3.3.2. Example

Niesters et al. used meta-regression to understand sex differences in opioid analgesia.^62^ These results indicated that there was no effect of age or study size on analgesia. However, they identified significantly greater effects of patient-controlled analgesia in women compared with men (effect size = 0.22, 95% CI: 0.02–0.42). Further analysis, which only included studies using morphine-based analgesia, showed even greater effect in women (effect size = 0.36, 95% CI: 0.17–0.56). With the increasing evidence of sex differences in pain mechanisms and processing,^58^ meta-regression could be an important tool to highlight sex differences in pain research.

### 3.4. Multivariate methods

Meta-analyses are often focused on a clinical topic with multiple correlated measures. The most appropriate way to analyse this type of data is through multivariate meta-analysis approaches. Multivariate meta-analysis simultaneously estimates the effect of multiple correlated outcomes.^15,45,71^ Due to the inherent variance included in multivariate methods, random-effects models are commonly used.^45,46,76^ Classic examples of multivariate analysis include assessing both the systolic and diastolic blood pressure or the sensitivity and specificity of a diagnostic test.^45^

#### 3.4.1. Considerations

Multivariate approaches are critical to consider when there are missing data or when the summarised effects depend on other correlated outcomes.^71,76^

Unfortunately, correlated outcomes are often assessed using multiple univariate analyses. This univariate approach can produce bias and overestimate the overall effect.^69^ This approach is also a common criticism of meta-analysis^19^ because it does not adequately assess the influence of multiple correlated outcomes on each other.^45,76^ Multivariate approaches help overcome this problem by accounting for the inherent dependence of certain outcomes in an analysis.^56^

#### 3.4.2. Example

Tagliaferri et al. analysed the contributions of multiple factors (pathological and psychological biomarkers) related to persistent nonspecific low back pain.^82^ They concluded that there were significant contributions of all studied biomarker categories to persistent low back pain (nervous system, spinal imaging, and psychosocial). However, psychosocial factors showed the greatest effect (Hedges g = 0.90, 95% CI: 0.69–1.10) compared with the nervous system (Hedges g = 0.31, 95% CI: 0.13–0.49) or spinal imaging measures (Hedges g = 0.55, 95% CI: 0.37–0.73). Due to the often complex and multifactorial nature of painful conditions, multivariate meta-analysis methods may elucidate important underlying factors that can facilitate patient stratification in various painful conditions.

### 3.5. Network meta-analysis

Network meta-analysis allows researchers to compare 3 or more interventions simultaneously by combining all of the available evidence both directly and indirectly across studies.^37^ By combining 2 different sets of interventions (ie, interventions A and B in study 1, and interventions B and C in study 2), it is possible to estimate the effects between 2 indirect interventions (interventions A and C). Network meta-analyses are beneficial for clinicians because they allow comparisons across the available evidence to rank the efficacy of different interventions for a clinical condition. This renders the findings more clinically relevant to the appropriate patient. It is particularly an advantageous method of meta-analysis because it allows for the comparison of interventions that have previously never been compared in primary studies.

#### 3.5.1. Considerations

Pairwise meta-analyses of the directly compared interventions should be performed before performing the network meta-analysis so that the statistical heterogeneity for each comparison can be directly evaluated. After this, the network meta-analysis model can be developed. Several models can be utilised for this: If there are no trials with multiple arms, meta-regression (described above) can be used; If multiarm trials are included, hierarchical models could be used within a Bayesian framework, or alternatively, a multivariate meta-analysis approach can be taken. Researchers should also prespecify how heterogeneity will be assessed within the model.

Network meta-analyses assume that there is consistency or agreement between the direct and indirect comparisons. However, this is not always the case and researchers must check for both global inconsistency across all comparisons and local inconsistency or “hotspots” within comparisons. If either of these are identified, it is important to closely examine the potential effect modifiers of studies within inconsistent loops. Network meta-regression models can also explore how the effect modifiers can affect the results. In addition, sensitivity analyses excluding studies that may be contributing to inconsistency can improve the robustness of the results.

#### 3.5.2. Example

Ho et al. performed a network meta-analysis comparing the effectiveness of various psychological interventions for chronic low back pain.^39^ This included 97 randomised controlled trials with 17 treatment nodes. They performed traditional pairwise meta-analyses for all direct comparisons and used random-effects network meta-analysis to combine the direct and indirect evidence. The mean rank and relative treatment rankings for each node were estimated, and the authors determined that the most highly ranked intervention for the primary outcome (physical functioning) at postintervention was cognitive behavioural therapy delivered with physiotherapy care (mean rank = 2.2, standardised mean differences = 1.01, 95% CI: 0.58–1.44).

### 3.6. Individual participant data methods

Individual participant data (IPD) meta-analysis is a method of obtaining and analysing raw individual level data from single studies instead of traditional group-level summary statistics.^70,81^ Individual participant data enables the identification of covariates or subgroups that traditional meta-analyses of aggregate data are not able to detect.^27,70,72,85^ As such, IPD meta-analysis is considered the benchmark for integrating data from clinical studies.^73,81^ With the increased need for personalised and stratified pain management, IPD meta-analysis has the potential to uncover the important and targeted treatment options that single randomised controlled trials are not powered to detect.^11,14,16,83^ This is particularly relevant for pain research because most clinical trials are not adequately powered to detect subgroup differences or identify relevant covariates. Recent advances in statistical modelling of IPD meta-analysis have shown promise and can be reviewed in detail here.^33,70,72,76^

#### 3.6.1. Considerations

Because IPD meta-analyses are more time and resource intensive, they should only be undertaken when traditional meta-analyses cannot adequately answer a clinical question.^72^ One such area for using IPD is to detect differences in treatment effects between individuals and account for covariates. With the often-disappointing results of potentially promising pain medications of the past few decades,^13,20,21,47,50^ IPD meta-analysis may provide important insights on how to identify significant subgroup differences in treatment effects. However, IPD meta-analyses are not always possible, and there are significant challenges with data sharing policies,^75^ data set harmonization,^1^ and obtaining full data sets.

#### 3.6.2. Example

Hayden et al. initially performed a systematic review and traditional meta-analysis that suggested that exercise therapy to be more effective in decreasing pain and improving function in patients with persistent low back pain^31^. However, this study used aggregate level data and was unable to identify which individuals may be more likely to benefit from exercise therapy. Then, the authors performed an IPD meta-analysis to identify different treatment effects of exercise among individual patients with persistent low back pain^32^. The overall IPD meta-analysis for persistent low back pain suggested that exercise was more beneficial than usual care or no treatment on pain at short-term follow-up (mean effect = −10.7, 95% CI: −14.1 to −7.4). This review also identified potential novel covariates of participants who may respond more favourably to an exercise intervention for persistent low back pain, including not having heavy physical work demands, normal body mass index, and any medication use for low back pain. These covariates could be used in future research to assess a stratified treatment approach for subgroups of patients with low back pain.

### 3.7. Prevalence

Prevalence meta-analysis is used to estimate the frequency of a disease occurring within a predefined population.^5^ Prevalence meta-analyses, such as the Global Burden of Disease Study,^1^ are valuable tools for researchers, clinicians, and policymakers to better understand disease burden and therefore direct resources and research appropriately. There are a variety of considerations to make when conducting a prevalence meta-analysis: the choice of method, model, variance estimation technique, whether the prevalence proportions need to be transformed, and method of heterogeneity assessment.

#### 3.7.1. Considerations

Currently, there are no reporting guidelines for prevalence meta-analyses. This results in reviews of varying quality.^10^ The main challenge with undertaking a prevalence meta-analysis is assessing heterogeneity.^57^ Within prevalence studies, there is likely to be variation in the underlying population, case definition, disease severity, and other biases, and therefore, a random-effects model should be utilised. To address heterogeneity, reviewers should assess for covariates that may explain heterogeneity and stratify the results into appropriate subgroups or perform meta-regression.

Transformation of the prevalence proportions may be necessary to obtain confidence intervals that do not lie in extreme ranges and variances that do not result in the undue weighting of studies. The most commonly recommended transformation is the Freeman–Tukey double–arcsine, followed by the logit, log, and arcsine transformations.^10^

#### 3.7.2. Example

Murray et al.^59^ conducted a meta-analysis examining the prevalence of chronic pain in young adults. They examined possible sources of heterogeneity by classifying studies by location of chronic pain, demographic, geographic, and psychosocial factors related to chronic pain as well as study-level characteristics such as population type, sampling area, years of data collection, and assessment method. The authors calculated heterogeneity using the I^2^ statistic and the Q test, and they found a very high degree of heterogeneity, with prevalence rates of chronic pain in young adults ranging from 1% to 41%, I^2^ = 99%, Q(42) = 5473.3. There was high heterogeneity even when the results were stratified by pain subtype.

## 4. Implications

**Figure 3.**
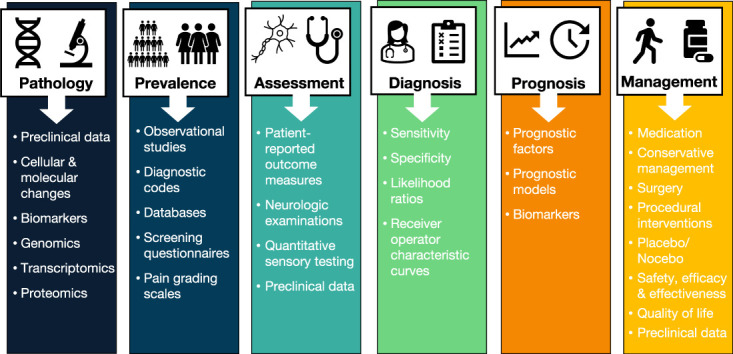
Examples of potential categories and data types that could be meta-analysed in the field of pain research.

The abundance and diversity of pain research creates unique opportunities to use meta-analysis in many areas (see examples in Fig. 3). These techniques are highly relevant for pain researchers and are currently being used to understand many aspects of pain. For example, there are several, large, multidisciplinary consortia actively collecting data to be meta-analysed.^1,1,68^ This enables large sample sizes and adequate power to detect significant effects for a range of biological and clinical variables, which cannot be identified in smaller studies.^43^

The complex pathological mechanisms of pain^6,48,86^ contribute to diverse and challenging clinical presentations. One approach to better understand and improve treatment for these pain phenotypes is through patient subgrouping, ie, stratification.^87^ Examples of data for patient stratification include clinical examination measures, QST, physiological and psychological factors, and molecular profiling.^83^ Meta-analysis can be a powerful tool to identify, organise, and analyse data to improve patient stratification.^38^

To continue advancing pain research, it is imperative to recognise what is currently known. Meta-analyses provide critical summaries of all available evidence to inform clinical practice and impact national and international guidelines^41,49,89^ and resource allocation. Although there are many different models and statistical considerations, meta-analysis is an important technique to understand and integrate these data. Meta-analyses can provide robust syntheses of published and unpublished data and can be planned prospectively through consortia and collaboration.

## 5. Conclusions

Meta-analysis can be used as a powerful tool to quantitatively synthesise important questions in pain research. In this review, we have highlighted several models and statistical methods to consider for the selection and interpretation of a meta-analysis. Although careful methodological consideration must be taken, meta-analyses can provide important summaries to facilitate scientific discovery and clinical advancement in pain research.

## Disclosures

The authors have no conflicts of interest to declare.
